# Marijuana associated with three-fold risk of death from hypertension

**Published:** 2017

## introduction

Marijuana use is associated with a three-fold risk of death from hypertension, according to research published recently in the European Journal of Preventive Cardiology.

‘Steps are being taken towards legalisation and decriminalisation of marijuana in the United States, and rates of recreational marijuana use may increase substantially as a result’, said lead author Barbara A Yankey, a PhD student in the School of Public Health, Georgia State University, Atlanta, US. ‘However, there is little research on the impact of marijuana use on cardiovascular and cerebrovascular mortality.’

In the absence of longitudinal data on marijuana use, the researchers designed a retrospective follow-up study of NHANES (National Health and Nutrition Examination Survey) involving participants aged 20 years and older. In 2005–2006, participants were asked if they had ever used marijuana. Those who answered ‘yes’ were considered marijuana users. Participants reported the age when they first tried marijuana and this was subtracted from their current age to calculate the duration of use.

Information on marijuana use was merged with mortality data in 2011 from the National Centre for Health Statistics. The researchers estimated the associations of marijuana use and duration of use with death from hypertension, heart disease and cerebrovascular disease, controlling for cigarette use and demographic variables including gender, age and ethnicity. Death from hypertension included multiple causes such as primary hypertension and hypertensive renal disease.

Among a total of 1 213 participants, 34% used neither marijuana nor cigarettes, 21% used only marijuana, 20% used marijuana and smoked cigarettes, 16% used marijuana and were past-smokers, 5% were past-smokers and 4% only smoked cigarettes. The average duration of marijuana use was 11.5 years.

Marijuana users had a higher risk of dying from hypertension. Compared to non-users, marijuana users had a 3.42-times higher risk of death from hypertension and a 1.04 greater risk for each year of use. There was no association between marijuana use and death from heart disease or cerebrovascular disease.

Ms Yankey pointed out that there were limitations to the way marijuana use was estimated. For example, it cannot be certain that participants used marijuana continuously since they first tried it.

She said: ‘Our results suggest a possible risk of hypertension mortality from marijuana use. This is not surprising since marijuana is known to have a number of effects on the cardiovascular system. Marijuana stimulates the sympathetic nervous system, leading to increases in heart rate, blood pressure and oxygen demand. Emergency rooms have reported cases of angina and heart attacks after marijuana use.’

‘We found higher estimated cardiovascular risks associated with marijuana use than cigarette smoking’, said Ms Yankey. ‘This indicates that marijuana use may carry even heavier consequences on the cardiovascular system than that already established for cigarette smoking. However, the number of smokers in our study was small and this needs to be examined in a larger study.’

‘Needless to say, the detrimental effects of marijuana on brain function far exceed that of cigarette smoking’, she added. Ms Yankey said it was crucial to understand the effects of marijuana on health so that policy makers and individuals could make informed decisions.

She said: ‘Support for liberal marijuana use is partly due to claims that it is beneficial and possibly not harmful to health. With the impending increase in recreational marijuana use it is important to establish whether any health benefits outweigh the potential health, social and economic risks. If marijuana use is implicated in cardiovascular diseases and deaths, then it rests on the health community and policy makers to protect the public.’

